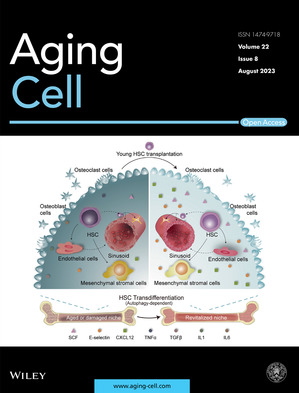# Featured Cover

**DOI:** 10.1111/acel.14010

**Published:** 2023-10-09

**Authors:** Na Yuan, Wen Wei, Li Ji, Jiawei Qian, Zhicong Jin, Hong Liu, Li Xu, Lei Li, Chen Zhao, Xueqin Gao, Yulong He, Mingyuan Wang, Longhai Tang, Yixuan Fang, Jianrong Wang

## Abstract

Cover legend: The cover image is based on the Research Article *Young donor hematopoietic stem cells revitalize aged or damaged bone marrow niche by transdifferentiating into functional niche cells* by Na Yuan et al., https://doi.org/10.1111/acel.13889